# Characterization of a Mutant Deficient for Ammonium and Nitric Oxide Signalling in the Model System *Chlamydomonas reinhardtii*

**DOI:** 10.1371/journal.pone.0155128

**Published:** 2016-05-05

**Authors:** Emanuel Sanz-Luque, Francisco Ocaña-Calahorro, Aurora Galván, Emilio Fernández, Amaury de Montaigu

**Affiliations:** Departamento de Bioquímica y Biología Molecular, Campus de Rabanales, Universidad de Córdoba, Córdoba, Spain; University Paris South, FRANCE

## Abstract

The ubiquitous signalling molecule Nitric Oxide (NO) is characterized not only by the variety of organisms in which it has been described, but also by the wealth of biological processes that it regulates. In contrast to the expanding repertoire of functions assigned to NO, however, the mechanisms of NO action usually remain unresolved, and genes that work within NO signalling cascades are seldom identified. A recent addition to the list of known NO functions is the regulation of the nitrogen assimilation pathway in the unicellular alga *Chlamydomonas reinhardtii*, a well-established model organism for genetic and molecular studies that offers new possibilities in the search for mediators of NO signalling. By further exploiting a collection of *Chlamydomonas* insertional mutant strains originally isolated for their insensitivity to the ammonium (NH_4_^+^) nitrogen source, we found a mutant which, in addition to its ammonium insensitive (AI) phenotype, was not capable of correctly sensing the NO signal. Similarly to what had previously been described in the AI strain cyg56, the expression of nitrogen assimilation genes in the mutant did not properly respond to treatments with various NO donors. Complementation experiments showed that *NON1* (*NO N**itrate 1*), a gene that encodes a protein containing no known functional domain, was the gene underlying the mutant phenotype. Beyond the identification of *NON1*, our findings broadly demonstrate the potential for *Chlamydomonas reinhardtii* to be used as a model system in the search for novel components of gene networks that mediate physiological responses to NO.

## Introduction

Nitric oxide (NO) is a signalling molecule whose presence and activity have been reported in a myriad of species belonging to almost all kingdoms of life. The pervasiveness of NO activity across a remarkable diversity of organisms illustrates how this signal has repeatedly been used throughout evolution to regulate many aspects of development and physiology. NO also stands out for the range of biological processes that it controls within the same organism. In *Arabidopsis thaliana* for example, a model plant species in which the functions of NO have been extensively studied, many processes are affected by NO levels such as the response of the immune system to pathogen attacks [1,2], developmental transitions from vegetative to reproductive phases [3], or gas exchange between the leaf and the atmosphere through the control of stomata opening [4,5]. The number of traits reported to be controlled by NO is increasing, yet our understanding of the genetic and mechanistic basis of how NO regulates these traits is limited. In comparison with chemical approaches consisting in describing physiological responses to the application of NO donors or scavengers, attempts to identify genes and proteins directly implicated in mediating the NO signal are relatively uncommon [6]. Unravelling the molecular events that lead from NO to specific physiological responses remains an important challenge, particularly in complex multicellular systems, and alternative strategies might be beneficial to bridge the gap between the signalling molecule and the phenotype.

To circumvent some of the issues associated with the identification of genes that act downstream of NO, a possibility is to export NO research to simple model systems suitable for high throughput genetic studies. In this sense, the recent implication of NO in the regulation of the nitrogen assimilation pathway in the model unicellular alga *Chlamydomonas reinhardtii* (*Chlamydomonas* hereafter) offers new possibilities in the search for mediators of NO signalling [7–9]. *Chlamydomonas* is a well-established organism for genetics and molecular biology [10], and over the years *Chlamydomonas* research has led to the identification of genes whose functions were first shown to be important for the alga, but later shown to be conserved in plants or humans [11–13]. The *Chlamydomonas* genome shows conservation with the genomes of organisms from both the plant and the animal kingdoms [1,2,12], meaning that this alga has a unique potential for the discovery of genes that could be of interest to very diverse fields of research. And even when genes of a particular pathway are not conserved from *Chlamydomonas* to other species, similar regulatory features can be retained in distantly related organisms. NO was demonstrated to repress nitrate assimilation in the alga [3,7,8], and although the gene that mediates this response is not conserved in plants, independent groups have shown that NO does repress nitrate assimilation in different plant species [4,5,7,14–17].

NO research in *Chlamydomonas* goes back to a study in which the authors intended to understand how NO was synthesized [6,18], a complex biological problem that is still under investigation in photosynthetic eukaryotes [7–9,19–21]. Since then, there have been several reports describing the major role of NO in regulating various components of the nitrogen assimilation pathway at the transcriptional and post-transcriptional levels [7,8,10]. Nitrate (NO_3_^-^) and ammonium (NH_4_^+^) are the two inorganic sources of nitrogen that most organisms are able to assimilate, but in natural environments nitrate is usually the available form [11–13,22] and its scarcity in soils is a limiting factor for the productivity of many cultivated crops. Genes of the nitrogen assimilation pathway are strikingly conserved between *Chlamydomonas* and plants, justifying why *Chlamydomonas* was adopted years ago as a model to study how the nitrogen pathway is regulated at the molecular level [13]. In the presence of both nitrogen sources *Chlamydomonas* preferentially assimilates ammonium which, once inside the cell, acts as a signal to repress the expression and inhibit the activity of nitrogen transporters and of enzymes that catalyse the reduction of nitrate [9]. NO was initially reported to be involved in regulating the nitrogen pathway by mediating the ammonium-dependent transcriptional repression of nitrate reductase (*NIA1*), of the nitrate transporter *NRT2*.*1*, and of the ammonium transporters *AMT1*.*1* and *AMT1*.*2* [7]. It was then shown to directly regulate ammonium and nitrate transporter activities as well as nitrate reductase (NR) activity independently of their transcription [8]. The mechanism through which NO inhibits NR activity was finally shown to involve the truncated hemoglobin THB1 [23–25]. Growing evidence therefore suggests that NO is a major regulator of nitrogen assimilation in *Chlamydomonas*.

The connection between NO and nitrogen assimilation was originally established through a mutant screen that aimed to identify novel regulators of *NIA1* transcription [26]. A fusion of the *NIA1* promoter to the arylsulfatase (*ARS*) reporter gene allowed selecting for ammonium insensitive (AI) insertional mutants in which ARS activity was still detectable when ammonium was present in the medium, condition under which the *NIA1* promoter is inactive in the wild type. The ARS enzyme is progressively excreted outside the cell into the medium where its activity is detectable with a simple assay that is well suited for the high throughput screening of thousands of mutants. A PCR method was designed to isolate the DNA sequences flanking the insertions [27], and a list of approximately 20 candidate genes (hereafter called the AI candidate list) for the ammonium-mediated repression of *NIA1* was established. One of these candidates was the NO-dependent soluble guanylate cyclase (GC) CYG56 [7], an enzyme that synthesizes the second messenger cGMP from GTP, and whose activity significantly increases upon binding of NO to its heme domain. The NO-GC-cGMP signal transduction cascade has thoroughly been described in different systems as a means of conveying information from NO by raising intracellular levels of cGMP. The use of the cyg56 mutant as a genetic tool together with chemicals that increase or reduce intracellular concentrations of NO or cGMP demonstrated the implication of these signalling molecules and of GC activity in ammonium mediated repression of nitrate assimilation [7].

The central position of *CYG56* in ammonium sensing was further strengthened after a large scale expression study revealed that, in addition to the regulation of CYG56 enzymatic activity by NO, the transcriptional regulation of the *CYG56* gene was also contributing to the control of the nitrogen pathway [28]. This large-scale study was designed to extensively analyse, in different genotypes and conditions, the expression of six candidate genes for ammonium sensing selected from the original AI candidate list. The goal was to identify similar regulatory features between genes that would support their implication in a common transcriptional network. The analysis identified the cysteine rich domain-containing protein *CDP1* as a novel regulator of nitrogen assimilation, and revealed that *CYG56* and *CDP1* were regulated in a similar way [28]. Both genes were upregulated by ammonium and, more importantly, were downregulated in the 54.10 mutant, which suggested that an unidentified upstream regulator of *CYG56* and *CDP1* was altered in this genotype. These findings generally consolidated the idea that the NO-CYG56 pathway was central to the regulation of nitrogen assimilation in response to ammonium, and identifying more of its genetic components will be necessary to better understand how it operates. The phenotype of cyg56 being only partial does suggest that additional genes implicated in NH_4_^+^ and NO signalling are still to be found, and some of these genes might figure amongst the AI candidate list from which *CYG56* was identified.

The aim of this work was to identify novel genes that act downstream of NH_4_^+^ and NO, and whose function is related to the NO-CYG56 pathway. Basing our strategy on the finding that the regulation of *CYG56* expression is part of a transcriptional network that senses ammonium, we sought to identify genes co-regulated with *CYG56* amongst a set of selected candidate genes for ammonium sensing. A subset of six candidates are analysed for the first time in this study and complement the existing subset of six genes that included *CYG56* and *CDP1* [28]. Extensive analysis of gene expression in different genotypes and conditions allowed to specifically search for genes whose transcript levels strongly correlated with transcript levels of *CYG56*. Three genes, all belonging to the new subset, responded to this criterion, and one of them was considered of particular interest as it was severely downregulated in the 54.10 mutant. The gene encodes a protein that contains no known functional domains, but the N terminal part of the predicted amino acid sequence shares homology with proteins of other algae. Detailed phenotypic analyses of the corresponding mutant confirmed its partial ammonium insensitivity, and also revealed that the mutant was insensitive to the application of NO donors. Complementation by transformation validated the identity of the candidate gene and of the co-expression approach, and the gene was named *NON 1* (*NO N**itrate 1)*. Thus, by searching for components of the NO-CYG56 pathway, we have isolated the second *Chlamydomonas* mutant impaired in NO and NH_4_^+^ sensing.

## Materials and Methods

### Strains and conditions

**Fig 1:**
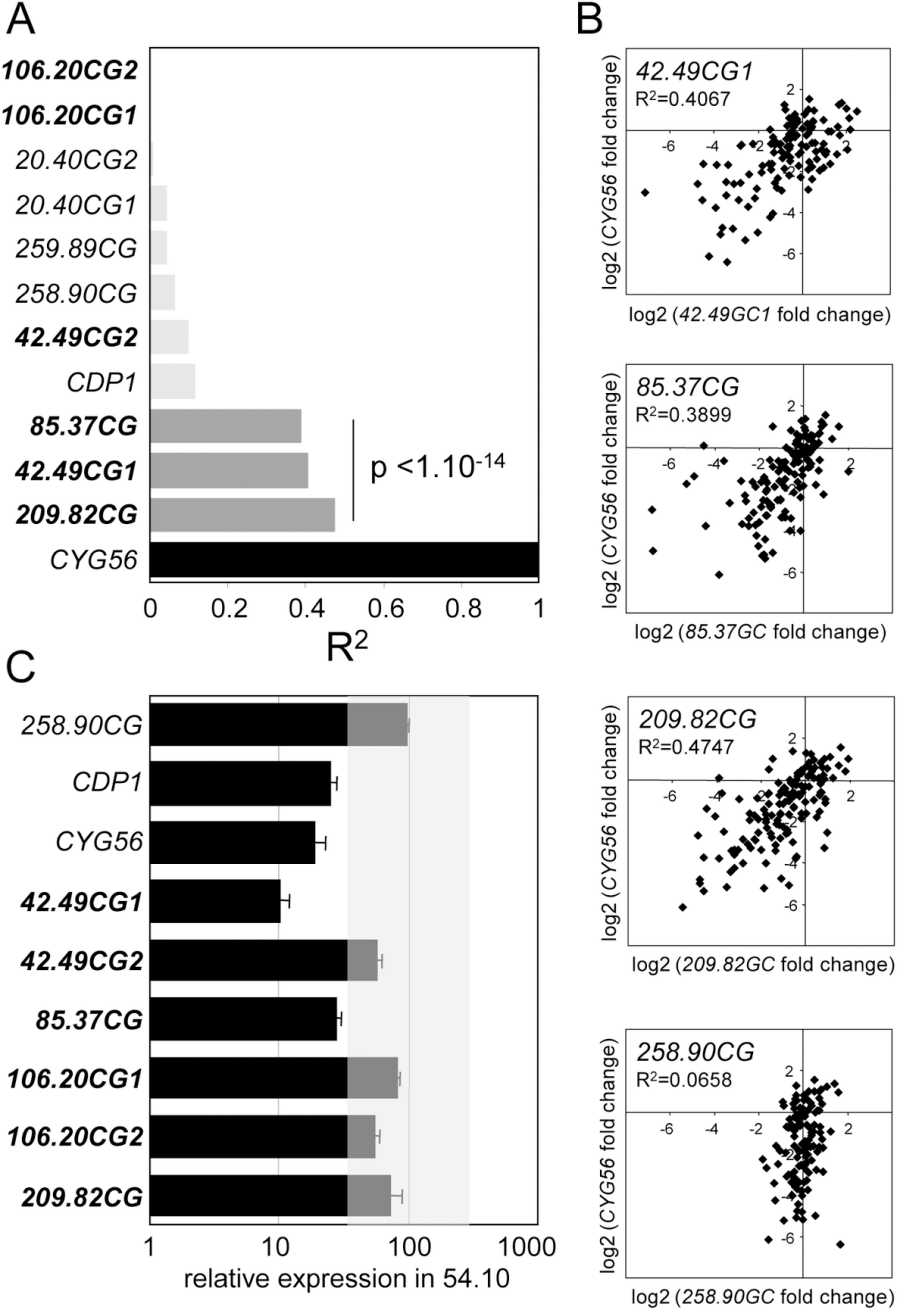
Identification of genes co-regulated with *CYG56*. (**A**) Pairwise correlations between *CYG56* expression levels and the expression levels of candidate genes for ammonium signalling. The squared correlation coefficient (R^2^) and the p values were determined with the Pearson test. 1 indicates a perfect correlation, as illustrated by the correlation of *CYG56* expression with itself (black bar). 0 indicates the absence of correlation. The three most significant correlations are indicated in dark grey. (**B**) Scatter plots showing the data distribution of the three most significant correlations detected in (A). The scatter plot showing *CYG56* expression levels plotted against *258*.*90CG* expression illustrates a negative result. (**C**) Expression of candidate genes in the 54.10 mutant. 54.10 was grown in four nitrogen contexts and harvested at four times points per condition (*see* Methods), and mean relative expression levels were calculated and presented using the same rationale than in a previous report [28]. Each mean was determined with the 16 data points so that it would reflect the general behaviour of a gene in the mutant and be robust to occasional misregulation patterns of a gene in a particular condition. A threefold cut off (shaded area) is used to highlight the most significant misregulation patterns. *CYG56* and *CDP1* are shown for comparison, and *258*.*90CG* as an illustration of a negative control. The genes analysed for the first time in this study are shown in bold characters.

The AI mutants were generated after insertional mutagenesis of the parental strain 704 (cw15 arg7^+^ *NIA1*::*ARS* mt^+^) [29] and were selected for resistance to the antibiotic paromomycin and for ARS activity in the presence of nitrate and ammonium [24]. The resulting genotype of these mutants was cw15 arg7^+^ *NIA1*::*ARS RBCS2*::*APHVIII* mt^+^. The AI mutants were given a name defined by two numbers [26], typically 42.49. The first number corresponds to the pool from which the mutant was isolated. The second number indicates the position of the mutant in the pool. Chlorate Sensitive in the presence of Ammonium (CSA) mutants N10 and N24 had a similar phenotype but were obtained with a different screen [30,31]. The samples used for the experiment presented in Fig 1 were obtained from the AI mutants cyg56, cdp1, 20.40, 54.10, 258.90 and 259.89, and from the CSA mutants N10 and N24 [28].

**Fig 2:**
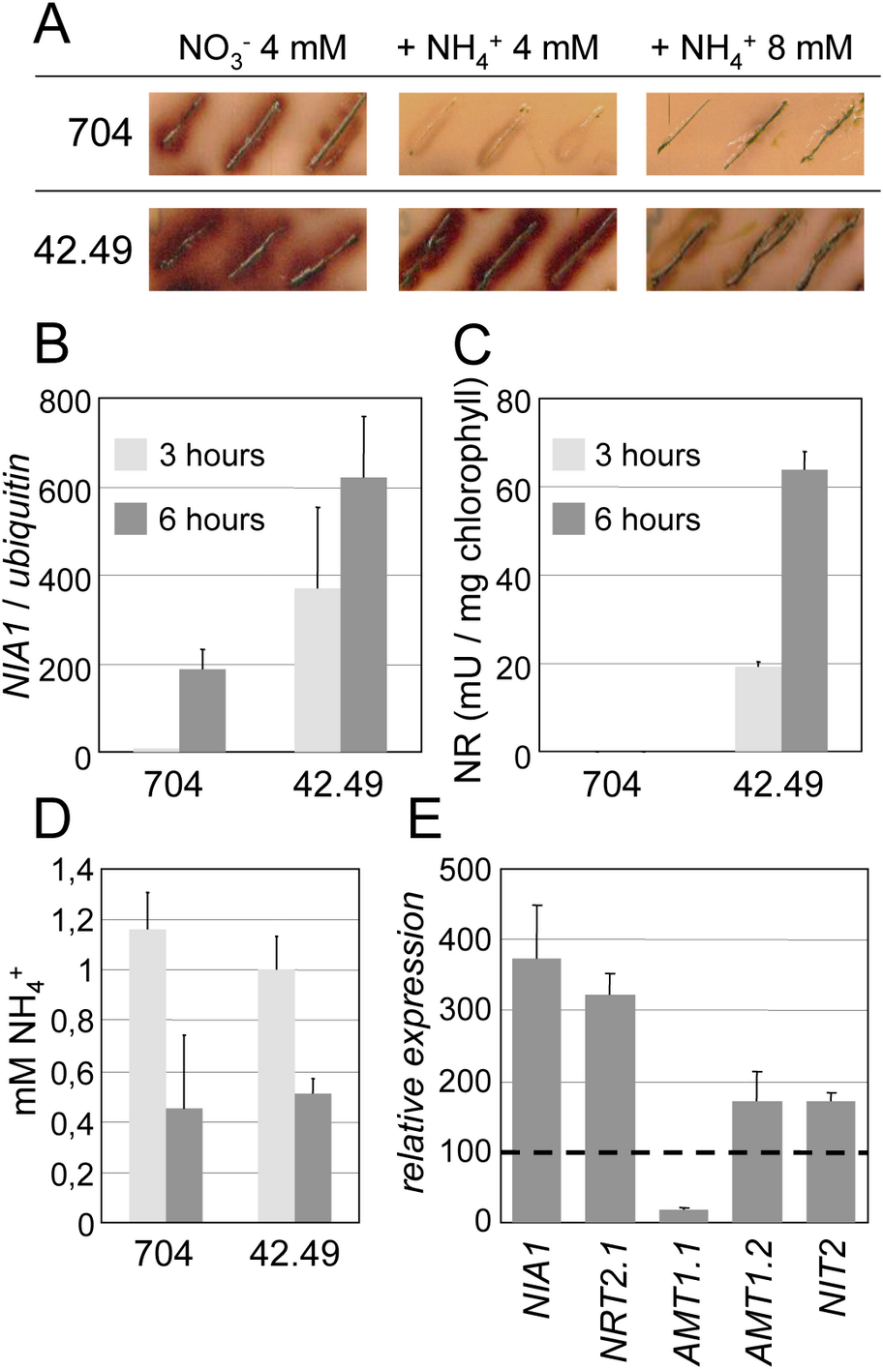
Phenotype of the 42.49 mutant. (**A**) Arylsulfatase (ARS) activity in the parental strain 704 and in the 42.49 mutant after four days on solid medium containing either 4 mM NO_3_^–^ as the sole nitrogen source, or NO_3_^–^ supplemented with NH_4_^+^ at the indicated concentrations. Both 704 and 42.49 strains bear a copy of the *ARS* gene fused to the *NIA1* promoter, so that ARS activity in the presence of NH_4_^+^ reveals that the promoter is not fully sensitive to NH_4_^+^ repression. (**B**) *NIA1* transcript abundance was quantified by qRT PCR in 704 and 42.49 strains after 3 and 6 hours in medium containing 4 mM NO_3_^–^ + 1 mM NH_4_^+^. The data were obtained from three technical replicates of two biological samples, and the error bars represent the standard deviation. (**C**) NR activity was determined in cell extracts of 704 and 42.49 strains in the same conditions than in (B). One mU of enzyme activity corresponds to the reduction of 1 nmol of substrate per minute. These results are representative of three independent biological replicates. (**D**) The residual concentration of NH_4_^+^ that remained in the medium was determined in parallel to the NR activity assay described in (C). (**E**) In an independent experiment from (B), transcript abundance of *NIA1*, *NRT2*.*1*, *AMT1*.*1*, *AMT1*.*2* and *NIT2* was determined in 704 (wt) and 42.49 after 6 hours in medium containing NO_3_^−^4 mM and NH_4_^+^ 1 mM. Results are expressed in % relative to the wild type. The presence of NH_4_^+^ was checked but not quantified.

All experiments except the ARS test (Fig 2A) were performed with liquid cultures of *Chlamydomonas* cells. The different strains were first grown in minimum medium [32] containing 8 mM of ammonium until the cell cultures reached exponential growth. Cells were then centrifuged and washed several times in medium without nitrogen before being transferred to the different induction media containing ammonium, nitrate, or other chemical compounds. Nitrate and ammonium were added in the forms of ammonium chloride or potassium nitrate. Growth chamber conditions were always constant light and 23°C. CO_2_ was provided by bubbling cultures with 5% (v/v) CO_2_-enriched air.

The concentrations of positive (nitrate) and negative (ammonium, NO, IBMX, A23187) signals used in each experiment were determined in previous reports [7,8,23,28]. They depended on whether the experiment was designed to measure short term or long term responses to the nutrients and chemicals, and on how fast the different compounds were consumed by the cells. In agreement with previous publications [24,28] it was also important to consider the balance between positive and negative signals. Ammonium-mediated repression of *NIA1*, for example, does not only depend on absolute amounts of ammonium but also depends on how much nitrate is present in the medium. The phenotype of the AI mutants being partial [28], genotypic effects are usually not detectable when cells are exposed to too much ammonium or not enough nitrate.

All these factors were taken into account to define the optimal concentration of the chemicals used for each assay. Long term ARS tests performed after several days of growth required high concentrations of nitrate and ammonium to make sure that the cells were still exposed to both nitrogen sources at the time the test was performed. A positive signal from the wild type in the presence of both nitrogen sources would simply indicate that all the ammonium had already been consumed. On the other hand, an excess of ammonium, typically 8 mM NH_4_^+^ for ARS experiments, would repress the activity of the marker even in the mutant (Fig 2A). For qRT and NR activity assays with both nitrogen sources, 1 mM ammonium and 4 mM of nitrate is the optimal condition to detect a short term response. For the chemical treatments we adjusted the concentration of nitrate to 100 μM because the chemicals are either less potent repressors of gene expression compared to ammonium or are used at lower concentrations.

### ARS activity

Cells were grown for four days on agar plates containing minimum medium supplied with different concentration of nitrate and ammonium. On the fourth day, the cells were removed from the surface of the agar plate with a razor blade, and the reaction mix including the ARS substrate was applied directly to the solid medium as previously described [33]. The plates were gently and continuously moved on a shaker so that the reaction mix would be evenly spread on the medium, which allowed for the ARS activity assay to be homogenous across the plate surface. The reaction was stopped after 15 min.

### NR activity

The method for in vitro determination of NR activity is based on protocols described in previous works [34,35]. First, 100μl of cells were lysed with 5 μl of 100% toluene to release the cytosolic NR enzyme in the reaction tube. One minute before starting the NR activity measurements, 1 mM of the electron acceptor ferricyanide was added to the tube to make sure that all the NR proteins were in their active oxidised state. The NR reaction was initiated by adding the electron donor benzyl viologen previously reduced with dithionite. The reaction was stopped by vigorous vortexing of the tubes leading to the immediate oxidisation of dithionite. Quantification of NR activity was determined by measuring how much nitrite, the product of the NR reaction, was present at the end of the assay [36].

### NH_4_^+^ concentration

The Nessler reagents were used to quantify the residual NH_4_^+^ concentration in the medium after NR activity assays and qRT-PCR experiments. Reagent A (25 μl) and reagent B (25 μl) were added to 500 μl of medium from which cells had been removed by centrifugation. OD was immediately measured at λ = 410 nm. Medium with 1 mM NH_4_^+^ starting concentration was diluted 10-fold to avoid saturation of the reaction.

### Complementation of the 42.49 mutant

The *NON1* genomic sequence was isolated from the Bacterial Artificial Chromosome (BAC) 33I20. A 7.8 Kb fragment including *NON1* was obtained after digestion of BAC 33I20 with the BamHI and SpeI enzymes, and was cloned in a pBluescript using the BamHI and SpeI restriction sites of the plasmid. In parallel, a bleomycin resistance cassette was amplified, sequenced and cloned in the pBluescript using the KpnI restriction motif. The 42.49 mutant was transformed with this construct and transformants were selected for resistance to bleomycin.

### Quantitative real-time PCR

In all experiments, cells were originally grown in medium containing NH_4_^+^ 8 mM as the sole nitrogen source until the cultures reached the exponential growth phase. Cells were then washed and transferred to the different induction medium for the indicated time periods After induction, cells were centrifuged (4000 g, 5 min) and lysed with 2% SDS in a lysis buffer composed of 100 mM Tris-HCl (pH 8.0), 400 mM NaCl, and 50 mM EDTA [37]. RNA was isolated by the phenol extraction method and precipitated with LiCl [38]. 1 μg RNA was reverse-transcribed using the oligo(dT) primer and the Superscript II reverse transcriptase (Invitrogen), following the recommendations of the manufacturer. For all the experiments except Fig 1, the qRT-PCRs were performed with an iCycler iQ real-time PCR detection system (Bio-Rad) using SYBR® Green I as a fluorescent dye (Molecular Probes). For Fig 1, PCRs were performed in an optical 384-well plate with an ABI PRISM® 7900 HT Sequence Detection System using the SYBR® Green Master Mix reagent (Applied Biosystems). All primers were designed using Primer Select (DNA Star Inc. v. 4.05) and are listed in S1 Table. The ubiquitin ligase was used as an internal standard to normalise the gene expression data [39].

### Co-expression analysis: description, rationale and treatment of the data

The samples were extracted from cell cultures of the wild type strain 704, six AI mutants and two CSA mutants (see Strains and Conditions) induced in medium containing NO_3_^-^ 4mM, NH_4_^+^ 8mM, NO_3_^-^ 4mM + NH_4_^+^ 8mM, or NO_3_^-^ 4mM + NH_4_^+^ 1mM [28]. AI and CSA mutants were isolated from different screens but have in common that they do not properly sense ammonium. Cells were harvested at four time points after the start of each induction (0.5, 1, 3 and 24 hours), raising the number of samples to 144. We originally quantified in these 144 samples the expression of six candidates for ammonium sensing (including *CYG56*) with the goal of identifying genes whose expression was altered in specific mutants. A reasonable assumption was that misregulation of a gene in a mutant might depend on the nitrogen context, which is why the strains were grown in medium containing different nitrogen sources. An advantage of diversifying conditions and time points was also to increase the number of samples that could then be used as “replicates” for specific purposes (Fig 2C; S1 Fig).

Prior to testing for correlations, the data were mean centred across conditions. The expression level of a gene determined in one strain and in one condition was normalized to the mean expression value of that gene calculated with the data from all the strains in that same condition. This mean-centring normalization strategy had been used in a previous report [28]. The aim of expressing the results as fold change relative to the mean was to limit the influence of the sampling condition on the results, so that positive correlations between *CYG56* and another gene would mostly be explained by the genotypic rather than the environmental variation (time point and nitrogen context). Thus, positive correlations would indicate that two genes were under the control of common upstream regulators and that they might be implicated in the same regulatory network. Correlations based solely on similar expression profiles in response to changing nitrogen conditions or to the time of sampling would not necessarily provide a strong indication of their implication in the same pathway.

After normalization, the data expressed as fold change relative to the mean were log2 transformed, as routinely performed in large-scale gene expression studies. The log-transformed data were then used to test for correlations with *CYG56* expression levels. Where applicable, the expression values of a gene measured in its corresponding mutant were not considered in the correlations, so that altered expression of a gene as a result of its interruption by the insertion would not skew the results. Finally, internal controls were used to strengthen the significance of the data. Weak correlations detected between *CYG56* expression and the expression of several other genes demonstrated that the positive results were not due to a technical artifact.

## Results

### Identification of genes co-regulated with *CYG56*

To search for genes co-expressed with *CYG56*, we took advantage of samples described in the *Materials and Methods* section and in another study [28]. Briefly, the samples were extracted from cell cultures of the wild type strain 704 and of eight mutants that were partially insensitive to ammonium. The strains were grown in four nitrogen contexts: NO_3_^-^ 4 mM and NH_4_^+^ 8 mM where nitrate assimilation genes are totally induced and repressed respectively, and NO_3_^-^ 4 mM + NH_4_^+^ 8 mM and NO_3_^-^ 4 mM + NH_4_^+^ 1 mM where two different levels of repression are observed [28]. Samples were harvested at four time points per condition (0.5, 1, 3 and 24 hours), raising the number of samples to 144 in the experiment overall. We had initially quantified the expression of a subset of six candidate genes for ammonium sensing in these samples, and *CYG56* transcript levels were shown to vary depending on the genotype and condition [28]. The current study builds on this result and on the success of the approach by further exploiting the information from the original AI candidate list [27] and by including in the experiment an additional subset of six candidate genes of interest. We then reanalysed the complete data set to search specifically for candidate genes whose expression strongly correlates with the expression of *CYG56*. Whereas the initial study had aimed to identify qualitative misregulation patterns of genes in mutants and conditions, the experiment presented here focuses exclusively on *CYG56*, and tests for the strength and statistical significance of quantitative pairwise correlations between the abundance of *CYG56* transcripts on one side and the transcript abundance of the candidate genes on the other. Importantly, the data normalization procedure allowed for positive correlations between *CYG56* and other genes to be mostly explained by the genotypic rather than the environmental variation (see Methods) [28].

The two subsets of candidate genes were both selected from the original AI candidate list, but their selection was based on different criteria. The initial subset of genes had been selected based on functional predictions consistent with a possible role in gene regulation, and based on clear alterations of their expression level as a direct consequence of their interruption by the insertion. In contrast, the new subset of genes was selected partly based on the strength of the phenotype of the mutant in which the gene was identified, and partly based on whether the expression of the candidate gene was detectable in the wild type strain. Not all genes from the original AI candidate list had detectable expression levels with standard qRT-PCR techniques, preventing their analysis in the co-expression experiment. The candidate genes of the new subset were identified in the AI mutants 42.49, 85.37, 106.20 and 209.82. For purposes of clarity, the genes were named after the name of the mutant in which they were identified followed by “CG” (Candidate Gene); e.g. *42*.*49CG1* was the first candidate gene interrupted by the insertion in mutant 42.49. Note that two genes were considered for analysis in each of the 42.49 and 106.20 mutants. In the case of 106.20, the predicted Peptidyl-Prolyl-cis-trans-Isomerase (PPIase) function of both *106*.*20CG1* and *106*.*20CG2* intriguingly coincided with the predicted PPIase function of the candidate gene identified in 258.90 (S2 Table). In the case of 42.49, the proximity of *42*.*49CG2* and *42*.*49CG1* in the genome was useful to strengthen the relevance of certain results described hereafter. Complementary data on the accession numbers of the genes, on their physical position on chromosomes according to the most recent version of the *Chlamydomonas* genome, and on the position of the insertions in the mutants are provided in S2 Table.

We found three genes that were strongly co-regulated with *CYG56*, all of which belonged to the new subset. The correlations between *85*.*37CG*, *42*.*49CG1* or *209*.*82CG* on one side, and *CYG56* on the other, yielded R^2^ values of approximately 0.4, far superior to the R^2^ values calculated with the data from the remaining eight genes (Fig 1A and 1B). Among the group of eight genes that did not correlate with *CYG56*, the genes *20*.*40CG1* and *258*.*90CG* were known to be stably expressed across genotypes and conditions [28]. The weak correlations of *CYG56* transcription with the transcription of *20*.*40CG1* and *258*.*90CG* was therefore fitting to predictions, and probably reflected some level of experimental noise. In contrast, the relationship between *CYG56* and *CDP1* expression was weaker than what might have been anticipated. Unlike *20*.*40CG1* and *258*.*90CG*, but similarly to *CYG56*, *CDP1* expression was not stable across conditions and was downregulated in mutant 54.10 [28]. The strength of the *CYG56* / *CDP1* correlation was nevertheless only slightly increased in comparison to the correlations observed for *20*.*40CG1* and *258*.*90CG*. This result showed that the qualitative and the quantitative approaches provided different information, because qualitative observations of similar misexpression patterns in the mutants, such as *CDP1* and *CYG56* downregulation in 54.10, was not sufficient for detecting strong correlations between the expression of two genes.

Conversely, strong positive correlations were not necessarily indicative of identical misexpression patterns in the mutants. Out of the three genes whose expression correlated with the expression of *CYG56*, only *42*.*49CG1* was severely downregulated in 54.10 (Fig 1C, S1 Fig). The stable expression of *209*.*82CG* in 54.10 and the milder downregulation of *85*.*37CG* relative *42*.*49CG1* in this genotype (Fig 1C) support the existence of different regulatory connections between *CYG56* and each of these genes. In the case of *CYG56* and *42*.*49CG1*, the correlation is in part, but not exclusively explained by the fact that both genes are strongly downregulated in 54.10. The correlation remains significant and amongst the highest in the data set if the data points from 54.10 are removed (R^2^ = 0.343, p = 2.24 10^−11^), meaning that common misexpression patterns of *42*.*49CG1* and *CYG56* in other samples also explain why these genes are detected as co-expressed in the analysis. In conclusion, we argue that the correlation approach revealed strong transcriptional bonds between pairs of genes, and the correlation between *42*.*49CG1* and *CYG56* as well as the strong downregulation of *42*.*49CG1* in the 54.10 mutant led us to focus further experiments on testing whether *42*.*49CG1* function was related to the NO-CYG56 pathway.

### *42*.*49CG1* mediates the repression of nitrate assimilation genes by NH_4_^+^

We performed a detailed analysis of the AI phenotype of the 42.49 mutant. ARS activity measurements on solid media containing nitrate (4 mM) and varying concentrations of ammonium showed that 4 mM of NH_4_^+^ repressed *NIA1* promoter activity in wild type 704 but not in 42.49 (Fig 2A). Consistent with the partial phenotype observed for all ammonium insensitive mutants reported to date [7,26,28], reduced ARS activity in the mutant grown in the presence of 8 mM compared to 4 mM of NH_4_^+^ demonstrated that the 42.49 mutant was not fully insensitive to ammonium. qRT PCR quantification confirmed the ARS experiments by showing that *NIA1* transcript levels were higher in the mutant compared to the wild type after 3 and 6 hours in the presence of 1 mM NH_4_^+^ (Fig 2B). This tendency was even more obvious with NR activity assays in which the activity of the enzyme was detected in 42.49 but not in 704 when NH_4_^+^ was present (Fig 2C). Control measurements of residual NH_4_^+^ in the medium supported that these results were not explained by one genotype consuming ammonium faster than the other (Fig 2D). Finally, genes of the nitrogen assimilation pathway were not all equally sensitive to the genetic defect of the 42.49 genotype (Fig 2E). *NRT2*.*1* expression in the mutant responded like *NIA1* to the presence of ammonium, whereas this response was mild for *AMT1*.*2* and *NIT2*, and absent for *AMT1*.*1*.

**Fig 3:**
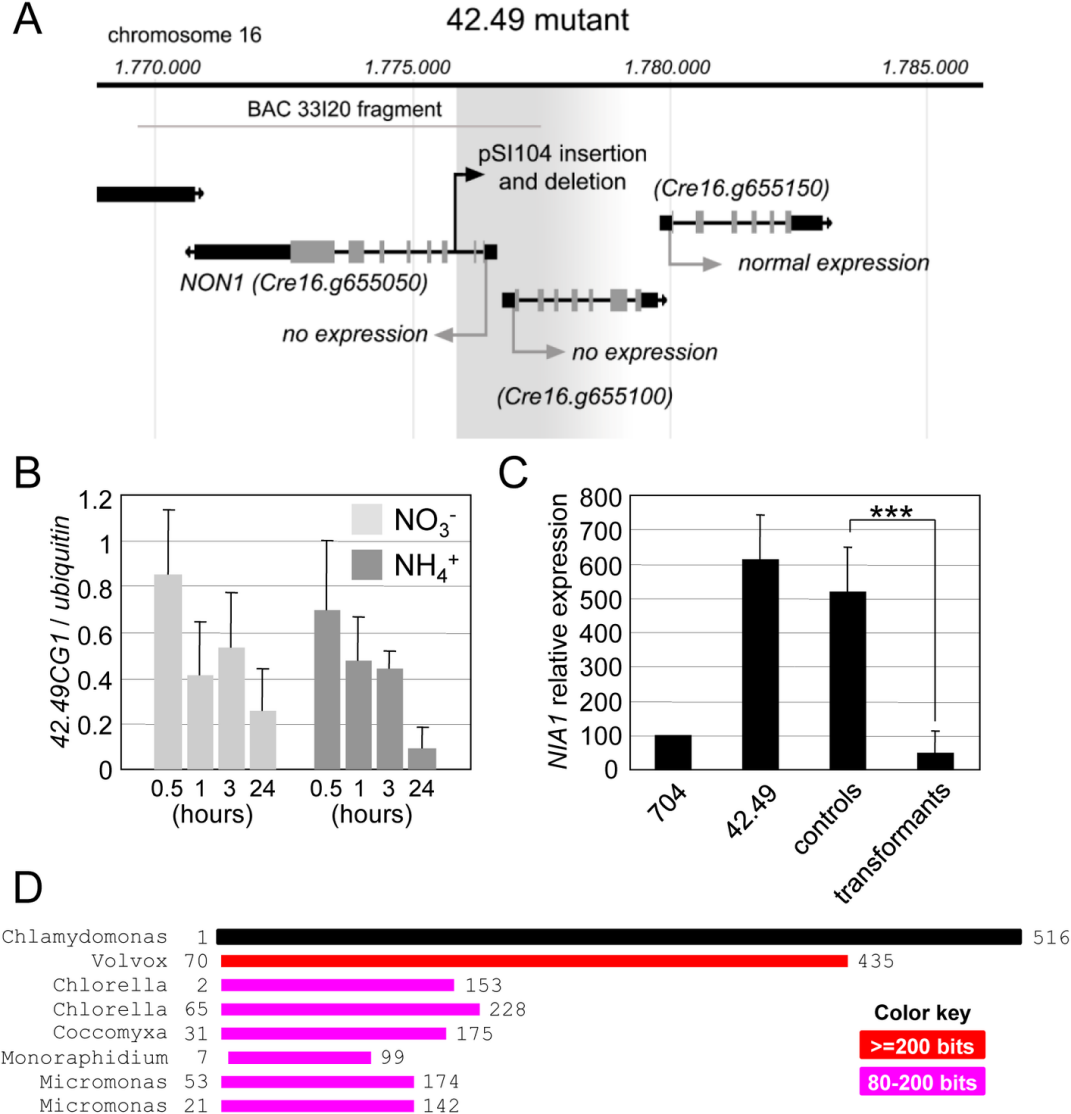
*NON1* (candidate gene *42*.*49CG1*) is the gene underlying the AI phenotype of the 42.49 mutant. (**A**) Position of the insertion in the 42.49 mutant. The insertion of the pSI104 plasmid took place at position 1775805 on chromosome 16, in the second intron of the *NON1* sequence (accession number Cre16.g655050). The two next downstream genes on the chromosome are also represented (accession numbers Cre16.g665100 and Cre16.g655150). Cre16.g665100 corresponds to the second designated candidate gene in this region (*42*.*49CG2*). Grey and black boxes indicate exons and UTRs, respectively. The grey arrows mark the start and orientation of the coding sequences. The black arrow indicates the position and orientation of the insert. The grey shaded area starting at the pSI104 position represents the deletion caused by the insertion, and the fading effect illustrates that the right border of the insertion has not been identified. The position of the 7.8 Kb fragment subcloned from BAC 33I20 and used for complementation is represented by a grey line. (**B**) *42*.*49CG1* expression was quantified in the wild type strain 704 grown in standard media containing 4 mM of NO_3_^-^ (light grey) or 8 mM of NH_4_^+^ (dark grey). Samples were harvested 30 minutes, 1 hour, 3 hours and 24 hours after induction in the two conditions. The means were calculated based on data from three technical replicates of two biological samples. Error bars represent the standard deviation. (**C**) Complementation of the AI phenotype with the *NON1* gene. The 42.49 mutant was transformed with a plasmid containing the *NON1* genomic DNA sequence, and *NON1* and *NIA1* transcripts were quantified by qRT PCR in the selected lines after 6 hours in medium containing NO_3_^−^4 mM and NH_4_^+^ 1 mM. Various lines were resistant to the antibiotic but did not express *NON1* (S2C Fig), and were used as negative controls. The histogram shows mean *NIA1* expression levels in positive transformants (n = 7) and negative controls (n = 5). Error bars represent the standard deviation. *** indicates p ≤ 10^−15^ with a Student t test (α = 0.05). (**D**) Graphical output of a BLAST analysis highlighting the conservation of the N terminal part of the NON1 protein with proteins of other algae. Sequence ID numbers of proteins from the different organisms are (from top to bottom): XP_002950714.1 (*Volvox carteri*), XP_005847655.1 (*Chlorella variabilis*), XP_005849673.1 (*Chlorella variabilis*), XP_005645512.1 (*Coccomyxa subellipsoidea*), KIY97900.1 (*Monoraphidium neglectum*), XP_002501227.1 (*Micromonas sp*. *RCC299*), XP_003062310.1 (*Micromonas pusilla* CCMP1545). Numbers indicate amino acid positions within the respective proteins.

The insertion in 42.49 has therefore interrupted a gene implicated in ammonium sensing and whose identity remains to be determined. qRT PCR and Southern blot experiments had demonstrated the presence of a single copy of the pSI104 plasmid in the 42.49 mutant [26]. The pSI104 insertion has occurred within the sequence of the *42*.*49CG1* gene and has caused a deletion of the 5’ end of its coding sequence, of its promoter region, and of an undetermined fragment of *42*.*49CG2* (Fig 3A). Despite the use of different strategies the genomic sequence flanking the right border of the insert could not be isolated (Fig 3A), but quantifying the expression of the genes in the immediate vicinity of the insertion revealed that only the expression of *42*.*49CG1* and *42*.*49CG2* was abolished in the mutant. Sequence analysis of these genes provided no information as to their potential regulatory role in the ammonium signalling chain (S2 Table), and their expression was not regulated by the nitrogen source in the wild type (Fig 3B, S2A Fig). What did provide information on a possible involvement in ammonium sensing was the comparison of the *42*.*49CG1* and *42*.*49CG2* correlations with *CYG56* transcript levels. While *42*.*49CG1* expression strongly correlated with *CYG56* expression in the data set, *42*.*49CG2* expression did not (Fig 1A). The absence of correlation between *42*.*49CG2* and *CYG56*, and the stable expression of *42*.*49CG2* in the AI mutants (S2B Fig) argues against *42*.*49CG2* being a candidate for ammonium sensing and supports *42*.*49CG1* as the gene underlying the mutant phenotype. Despite the proximity of the *42*.*49CG1* and *42*.*49CG2* transcriptional start sites (Fig 3A) and the possible existence of promoter elements shared between these genes, contrasting results of the correlation analysis must be due to regulatory motifs that contribute specifically to *42*.*49CG1* expression and that explain why this gene is co-regulated with *CYG56*.

To further strengthen that *42*.*49CG1* was the gene whose interruption was the cause of the AI phenotype, 42.49 was transformed with a 7.8 Kb fragment of BAC 33120 containing only the genomic sequence of *42*.*49CG1* (Fig 3A). The presence of this fragment in the transformants significantly reduced the expression of *NIA1* to levels that were comparable to, or lower than those detected in the wild type (Fig 3C). Reduced *NIA1* transcript levels were detected in 7 out of the 9 transformants that expressed the *42*.*49CG1* transgene (S2C Fig). Negative results obtained for the two transformants T8 and T9 could simply be due to complex insertion events not uncommon in *Chlamydomonas* insertion lines [40,41]. It is possible that sequence rearrangements at the insertion sites of T8 and T9 gave rise to the expression of a non-functional transgene. Two observations support that the results obtained with these transformants are misleading. First, T8 and T9 were the only transformants out of 14 (including controls) to display higher *NIA1* expression than 42.49 itself (S2C Fig). Second, the tendency of the insert to overcomplement in T1 to T7 regardless of the expression level of the transgene was in sharp contrast with the absence of complementation in T8 and T9 despite above average, and even abnormally high transgene expression levels (S2C Fig). Taken together, our experiments support that loss of *42*.*49CG1* function is the cause of the AI phenotype in 42.49 and that it might be implicated in the same regulatory network than *CYG56*.

### *NON1* mediates NO repression of nitrate assimilation genes

From this point onwards, the *42*.*49CG1* candidate gene will be renamed *NO N**itrate 1* (*NON1*) in reference to its function as a repressor of nitrate assimilation and as a mediator of NO signalling (see below). *NON1* (*Cre 16*.*g655050*) encodes a putative protein of 516 aa with no known functional domain (Phytozome, *Chlamydomonas reinhardtii* v5.5), and the N terminal part of the protein shares homology with the N terminal part of other algal proteins that have not been characterized (Fig 3D, S3 Fig). These observations strongly suggest that NON1 is a novel type of regulator found predominantly in algae, although it seems premature to exclude that NON1 function is not present in other taxa solely based on the absence of sequence homologies. More detailed studies could eventually lead to the discovery of genes that fulfil the same function, and the current work takes a first step in this direction by reporting the isolation and characterisation of the 42.49 mutant. *NON1* loss of function impairs nitrogen assimilation, and its expression strongly correlates with the expression of the NO-inducible guanylate cyclase *CYG56*. We therefore hypothesized that NON1 might be related to the NO-CYG56 pathway, and that its function may be required to convey the NO signal. If this hypothesis was correct, the 42.49 mutant should behave similarly to cyg56 in the sense that it should be partly insensitive to a rise in NO levels. We tested this idea by measuring how strongly genes of the nitrogen assimilation pathway were repressed in 42.49 after the application of chemical compounds to which cyg56 does not properly respond.

**Fig 4:**
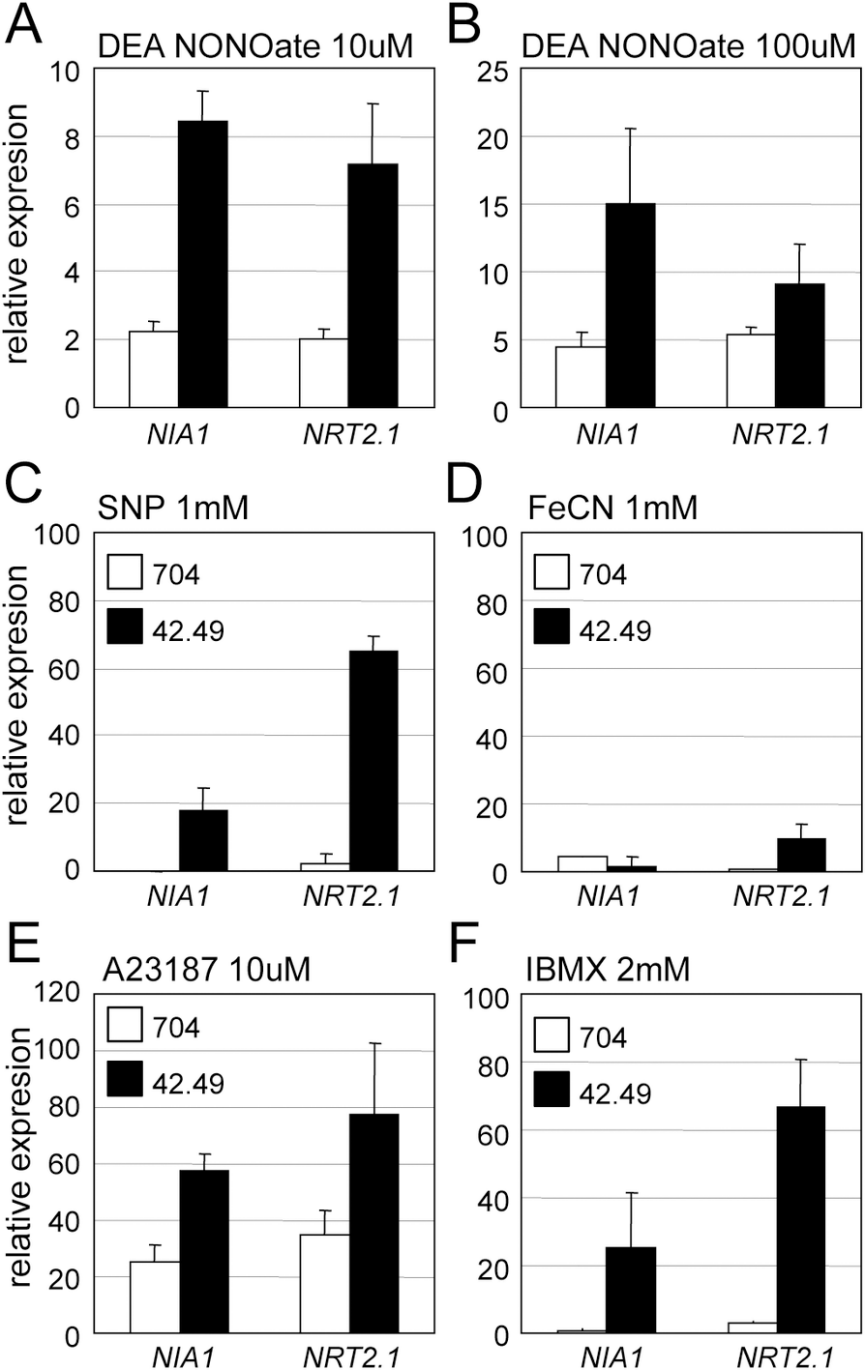
Repression of *NIA1* and *NRT2*.*1* in the 42.49 mutant is partially insensitive to treatments with NO donors, A23187 and IBMX. *NIA1* and *NRT2*.*1* transcript levels were quantified by qRT-PCR in the 704 parental and in the 42.49 mutant after treatment with (A) and (B) DEA NONOate, (C) and (D) SNP and FeCN chemical control, (E) A23187, and (F) IBMX. The strains were originally grown in medium containing NH_4_^+^ 8 mM as the sole nitrogen source until the cultures reached the exponential growth phase. Cells were then washed and transferred to the induction medium containing NO_3_^-^ 100 μM plus different chemicals at the indicated concentration. The NO_3_^-^ concentration used in this experiment was lower compared to previous experiments because, among other reasons, the chemicals are less potent repressors of gene expression than ammonium. These technical issues are discussed in the *Material and Methods* section. Samples were harvested 1 hour after treatment. Results are expressed in % relative to the untreated control.

These experiments demonstrated that NON1 was acting downstream of NO to repress *NIA1* and *NRT2*.*1*. NO donors DEA NONOate (DEA) and Sodium Nitroprusside (SNP) repressed *NIA1* and *NRT2*.*1* in the wild type, and this repression was partly relieved by the 42.49 mutation (Fig 4A–4D). SNP is a less potent NO donor than DEA which likely explains why the effect of *NON1* loss of function is stronger in response to this product. Ferricyanide (FeCN), a chemical analogue of SNP that does not release NO, also repressed the expression of *NIA1* and *NRT2*.*1* as previously described [7], but similar responses to FeCN of the wild type compared to the mutant supported that the 42.49 response to SNP treatments was specific to NO (Fig 4D). The partial insensitivity of 42.49 to NO donors put forward the similarities between 42.49 and cyg56, and was consistent with *NON1* function being associated with the NO-CYG56 pathway. cyg56 is also partially insensitive to A23187, a calcium ionophore that increases intracellular levels of Ca^2+^ and that represses the expression of *NIA1* and *NRT2*.*1* [7]. As observed in the cyg56 mutant, Ca^2+^-mediated (A23187) repression of *NIA1* and *NRT2*.*1* was partly relieved in 42.49 (Fig 4E). Like CYG56, NON1 therefore acts downstream of NH_4_^+^, NO and A23187 to repress genes of the nitrogen pathway, which together with the co-expression of *NON1* and *CYG56* suggests that the function of these genes could be intimately related. Independently of its connection to *CYG56*, our results suggest that *NON1* codifies a novel mediator of NO signalling.

CYG56 synthesizes cGMP in response to NO, and an increase in the intracellular concentration of cGMP induced by the phosphodiesterase inhibitor isobutylmethylxanthine (IBMX) compensates for loss of CYG56 activity in the cyg56 mutant [7]. Following the same rationale than above we tested the effect of IBMX on 42.49 cells, but contrary to what had been observed for cyg56 [7], *NIA1* and *NRT2*.*1* were partially insensitive to the presence of the compound (Fig 4F). Unexpectedly, we also found that IBMX stimulated the expression of *NON1* in the wild type, and that *NON1* expression was repressed by the guanylate cyclase (GC) inhibitors LY83,583 (6-anilino-5,8-quinolinedione) and ODQ (1H-[1,2,4]oxadiazolo-[4,3-a] quinoxalin-1-one) (S4 Fig). The transcriptional response of *NON1* to the chemicals was a good indication that *NON1* function was related to a pathway involving GC activity. The insensitivity of 42.49 to IBMX additionally supported that GCs including CYG56 could act upstream of *NON1* by regulating its transcription. Functional redundancy between more than 50 GC catalytic domains present in the genome of *Chlamydomonas* [12] most certainly explains why loss of CYG56 function in cyg56 is by itself not sufficient to alter *NON1* expression (S1A Fig). Until now, the data had shown that cyg56 and 42.49 responded similarly to NH_4_^+^, NO donors and A23187, but the experiments described here, particularly the response of 42.49 to IBMX, brought to light differences between the two mutants. These differences could imply that NON1 mediates NO signalling by acting downstream of GCs. Alternatively, they could also be considered as evidence for an NO independent function of NON1 on the regulation of gene expression (see Discussion).

**Fig 5:**
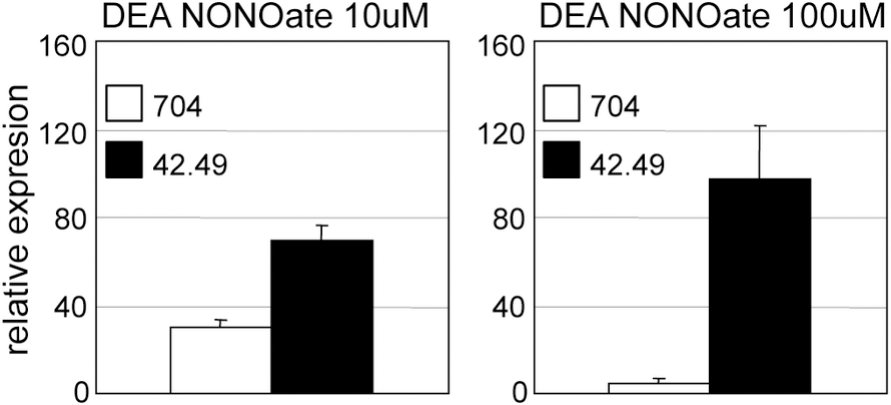
*AMT1*.*2* is highly insensitive to DEA NONOate in 42.49. Experiments were performed and results treated as in Fig 4. DEA NONOate was applied at the indicated concentrations.

One of the main proposals of this work is that *NON1* acts downstream of NO to repress gene expression, and this idea was reinforced after testing how molecular markers other than *NIA1* and *NRT2*.*1* were responding to NO in 42.49 (Fig 5). The experiments had focused on *NIA1* and *NRT2*.*1* because NO-mediated repression of these genes was known to be affected in cyg56, but other genes such as ammonium transporters are also repressed by NO in the wild type [7]. *AMT1*.*2*, whose response to ammonium was weakly affected in 42.49 (Fig 2E), was strikingly insensitive to the strong NO donor DEA (Fig 5), much more than *NIA1* and *NRT2*.*1* (Fig 4A and 4B). The specific response of *AMT1*.*2* to NO in 42.49 supports the involvement of *NON1* in conveying information from NO to the nitrogen assimilation pathway, and illustrates how NO-dependent regulatory mechanisms may differentially act on certain target genes. Deciphering NO-dependent transduction cascades will require characterizing multiple NO targets (e.g. *AMT1*.*2*) not only for demonstrating the implication of a gene (e.g. *NON1*) in NO signalling, but also for determining the specificity of its action.

## Discussion

This work describes the characterisation of 42.49, a *Chlamydomonas* mutant impaired in NH_4_^+^ and NO signalling. Our experiments collectively imply that the interruption of the *NON1* gene is the cause the 42.49 phenotype and that *NON1* is a novel mediator of the NO signal. The data more generally improve our understanding of the genetic network that causes the shutdown of nitrate assimilation in response to NH_4_^+^ and NO. To reach our conclusions, we initially sought to identify genes whose function could be associated with the NO-CYG56 pathway. A co-expression analysis identified *NON1* amongst a set of candidate genes originally described in mutants displaying the same phenotype than cyg56. Complementation by transformation showed that *NON1* was the gene underlying the 42.49 phenotype, and treatments with NO donors supported that *NON1* was necessary to properly sense NO. That *NON1* and *CYG56* were co-regulated and that they acted downstream of the same signals (NH_4_^+^, NO and Ca^2+^) to repress the same set of target genes at the least indicated that the physiological roles of *NON1* and *CYG56* were closely related. At the most, these data argued that *NON1* and *CYG56* were components of a single molecular route, and that their functions might be organised following a defined regulatory hierarchy.

The trend that emerges from the insensitivity of the 42.49 mutant to IBMX, and from the regulation of *NON1* by IBMX and GC inhibitors is that *NON1* could act downstream of GC activity. *CYG56* loss of function was not sufficient to alter *NON1* transcript levels, so the repression of *NON1* by LY83,583 and ODQ implied that *NON1* could be under the control of multiple GCs. The higher concentrations of ODQ compared to LY83,583 needed to repress *NON1* additionally implied that soluble and non-soluble GCs might not be equally important for the regulation of *NON1* expression (S4 Fig). LY83,583 is a general inhibitor of GC activity, whereas ODQ acts specifically on NO-inducible GCs such as CYG56. Considering the high concentrations of ODQ needed to repress *NON1* and the absence of effect of the cyg56 mutation on the expression of this gene, it cannot be excluded that NO-inducible GCs are not major regulators of *NON1* transcription and that *NON1* is preferentially regulated by the non-soluble forms of this enzyme. If this is the case, *NON1* transcriptional regulation by GCs would not be part of the NO signalling chain, and the roles of *NON1* downstream of NO and downstream of GC activity would be two independent functions. Finding the *CYG* genes that act redundantly with *CYG56* and studying the biochemical properties of the NON1 protein will be needed to determine whether or not NON1 mediates the NO signal by acting downstream of NO inducible GCs.

The position of *NON1* relative to *CYG56* in the pathway remains unresolved, but their co-regulation was a supplementary indication that NON1 function was related to CYG56 and to NO. The strength of the correlation between *NON1* and *CYG56* was partially explained by the downregulation of *NON1* and *CYG56* in the 54.10 mutant. No obvious candidate gene has yet been identified in 54.10, but its outstanding phenotype is probably caused by the interruption of a central regulator for ammonium sensing whose function might be to coordinate the transcription of *CYG56*, *NON1*, and maybe of other genes [28]. Coordinating the expression of these genes may be a way of optimizing the activity of the pathway by ensuring that sufficient quantities of the corresponding gene products are simultaneously present to cooperatively repress nitrogen assimilation. The generation of double mutants should help precisely define the regulatory hierarchies between *NON1*, *CYG56* and the gene interrupted in 54.10, but sexual reproduction in *Chlamydomonas* is intimately related to nitrogen assimilation, and no crosses could be obtained with the 42.49 genotype so far. Regardless of the difficulties to mate these strains, the use of the loss of function mutants as genetic tools has been instrumental for establishing regulatory connections between repressors of nitrate assimilation [28] (this work).

The characterisation of the AI mutants is revealing an unforeseen relationship between NO and the nitrogen assimilation pathway, and exploiting this genetic resource is leading to the discovery of potential mediators of NO signalling such as *NON1*. The co-regulation of *NON1* with *CYG56* was already an indication, but it is the insensitivity of 42.49 to NO donors, particularly the absence of *AMT1*.*2* repression in response to NO, that provided the strongest evidence for *NON1* being a mediator of the NO signal. 42.49 is the second AI mutant after cyg56 to be altered in its capacity to convey information from NO, and a question that arises from the characterization of these genotypes is whether NO and cGMP are the major signals through which NH_4_^+^ represses nitrate assimilation. The presence of more than 50 predicted catalytic GC domains in the genome of *Chlamydomonas* calls for more detailed studies of their individual functions. GCs do not seem to be so frequent in all photosynthetic eukaryotes and their physiological roles remain poorly understood. Nevertheless, independent experiments have shown that NO and cGMP can repress genes of the *Arabidopsis* nitrogen assimilation pathway [7,17,42]. NON1 and CYG56 may not be present in plants, but the regulatory logic of NO and cGMP being repressors of nitrogen genes seems to be evolutionarily conserved. The presence in other organisms of genes homologous to certain candidate genes from the original AI candidate list holds the promise of identifying novel components of NO signalling that are conserved across species.

To continue isolating mutants associated with NO signalling, an interesting strategy could be testing the sensitivity to NO donors of all the AI mutants. A drawback of this strategy, however, is that it will restrict the scope of the findings to the nitrogen assimilation pathway and prevent the identification of specialized NO sensors with broader physiological roles. More ambitious would be to directly screen for mutants insensitive to NO donors. Strictly speaking, *NIA1*::*ARS* could be used as a marker for the screen, although our results imply that the *NIA1* promoter might not be the optimal choice (Fig 5) and that the ARS reporter is probably not the most sensitive system. An attractive option to replace ARS is the Luciferase enzyme. Luciferase has been implemented in *Chlamydomonas* [43] and would be perfectly adapted for measuring responses to NO donors that rapidly release NO in the medium. Finally, the use of different approaches to monitor intracellular NO levels [44] could be a means of isolating mutants implicated in NO synthesis. With the optimization of the methodology to generate, screen, and isolate thousands of transformants, and with the efficiency of the molecular techniques designed to identify mutated genes of interest, insertional mutagenesis in *Chlamydomonas* has become an increasingly popular tool [26,41,45,46]. The exploitation of this resource represents an alternative to the strategies used in NO research until now, and will most certainly contribute to advances in our understanding of how this universal signalling molecule is perceived and synthesised.

## Supporting Information

S1 Fig Mean relative expression of candidate genes *42*.*49CG1*, *85*.*37CG*, and *209*.*82CG* in the eight mutant strains. Genotypes were grown in four nitrogen contexts and harvested at four times points per condition (see Materials and Methods). Mean relative expression levels were calculated and presented using the same rationale than in Fig 1D. A threefold cut off (shaded area) is used to highlight the most significant misregulation patterns.
(PDF)

S2 Fig Additional information on the complementation experiment. (**A**) *42*.*49CG2* expression was quantified in the wild type strain 704 grown in standard media containing 4 mM of NO_3_^-^ (light grey) or 8 mM of NH_4_^+^ (dark grey). Samples were harvested 30 minutes, 1 hour, 3 hours and 24 hours after induction in the two conditions. The means were calculated based on data from three technical replicates of two biological samples. Error bars represent the standard deviation. (**B**) Mean relative expression of candidate genes *42*.*49CG2* in the eight mutants. The data were treated as in Fig 1D, S1 Fig and as detailed in the *Methods*. (**C**) *NON1* and *NIA1* expression in 704, non1 and in individual transgenic lines (C1 to C5 and T1 to T9). Transgenics were generated by transforming non1 with a plasmid containing the *NON1* genomic sequence. Multiple lines resistant to the antibiotic were selected, and *NON1* and *NIA1* transcripts were quantified by qRT PCR in each line grown during 6 hours in medium containing NO_3_^−^4 mM and NH_4_^+^ 4 mM. The five lines that were resistant to the antibiotic but that did not express *NON1* were used as negative controls (lines C1 to C5). Two transformants (T8 and T9) were considered false positives because, although *NON1* expression was detected in these genotypes, they showed abnormal expression levels of *NIA1* and, in the case of T9, of *NON1* itself.
(PDF)

S3 Fig Alignment of the N terminal part of NON1 with homologous sequences of eight algae proteins. A graphical representation of the alignment is provided in Fig 3. Sequence ID numbers of proteins from the different organisms are (from top to bottom): XP_002950714.1 (*Volvox carteri*), XP_005847655.1 (*Chlorella variabilis*), XP_005849673.1 (*Chlorella variabilis*), XP_005645512.1 (*Coccomyxa subellipsoidea*), KIY97900.1 (*Monoraphidium neglectum*), XP_002501227.1 (*Micromonas sp*. *RCC299*), XP_003062310.1 (*Micromonas pusilla* CCMP1545). Numbers indicate amino acid positions within the respective proteins. Stars below the alignment indicate 100% conservation of the corresponding amino acids.
(PDF)

S4 Fig The influence on *NON1* expression of the phosphodiesterase inhibitor IBMX, and the guanylate cyclase inhibitors LY83,583 and ODQ. The 704 parental strain was grown on NH_4_^+^ 8 mM medium until the cell culture reached exponential phase, and the cells were washed and transferred to media containing (A) NO_3_^-^ 100 μM or (B) and (C) NO_3_^−^4 mM and NH_4_^+^ 1 mM. The different chemicals were applied at the indicated concentrations and samples were harvested 1 hour after treatment for quantification of *NON1* expression.
(PDF)

S1 Table List of primers used in this study. (DOCX)

S2 Table Details of genes analysed in Fig 1. (DOCX)
