# Supplementary figures and images for: Characterization of a Mutant Deficient for Ammonium and Nitric Oxide Signalling in the Model System Chlamydomonas reinhardtii
Source: PLoS One. 2016 May 5;11(5):e0155128. doi: 10.1371/journal.pone.0155128 (PMC4858171; doi:10.1371/journal.pone.0155128)

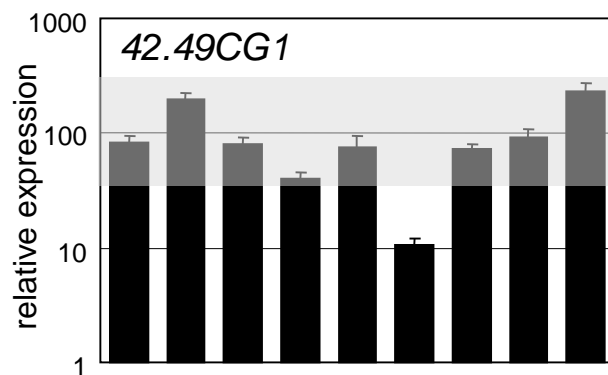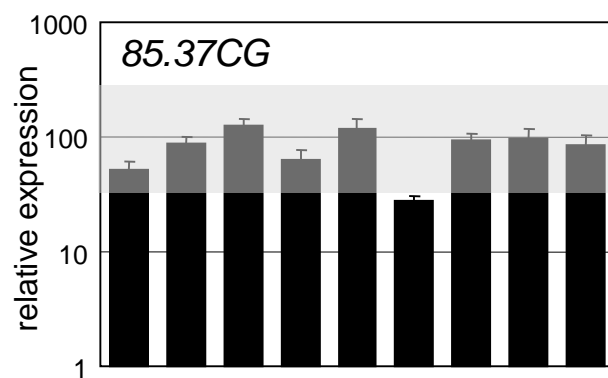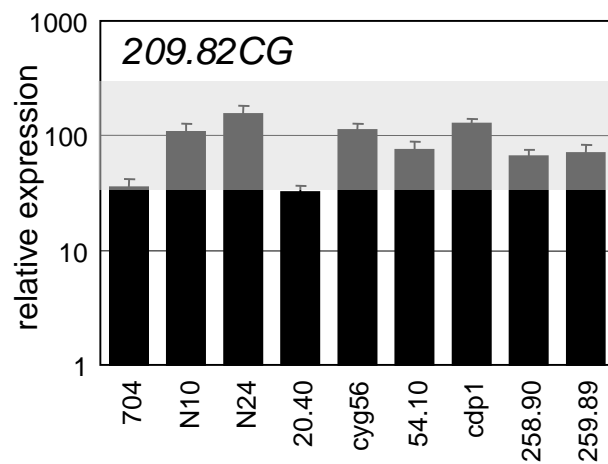

Supplement: S1 Fig — Genotypes were grown in four nitrogen contexts and harvested at four times points per condition (see Materials and Methods). Mean relative expression levels were calculated and presented using the same rationale than in Fig 1D. A threefold cut off (shaded area) is used to highlight the most significant misregulation patterns. (PDF) [file pone.0155128.s001.pdf]

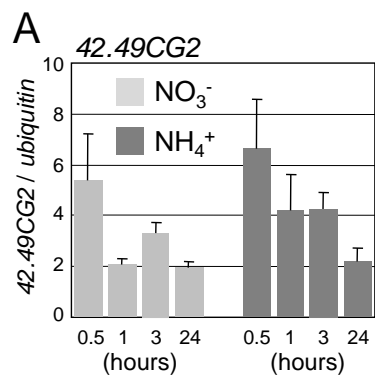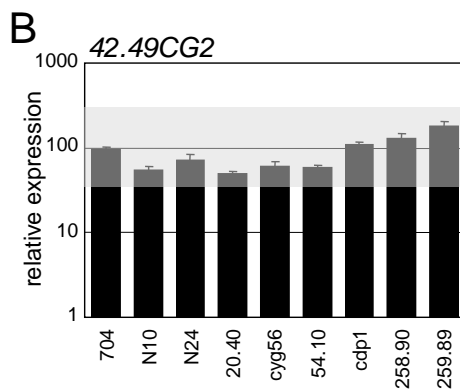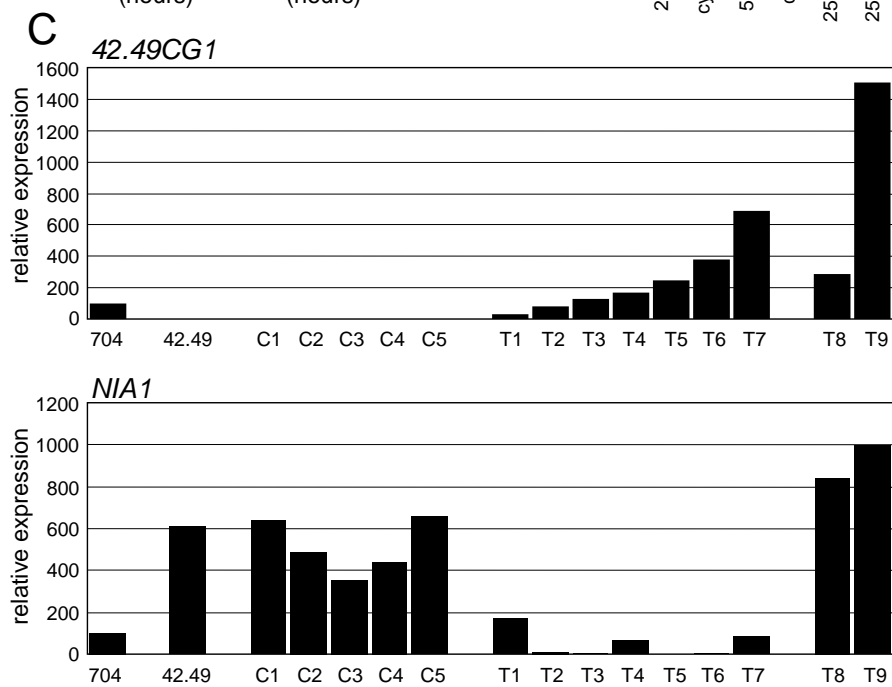

Supplement: S2 Fig — (A) 42.49CG2 expression was quantified in the wild type strain 704 grown in standard media containing 4 mM of NO3- (light grey) or 8 mM of NH4+ (dark grey). Samples were harvested 30 minutes, 1 hour, 3 hours and 24 hours after induction in the two conditions. The means were calculated based on data from three technical replicates of two biological samples. Error bars represent the standard deviation. (B) Mean relative expression of candidate genes 42.49CG2 in the eight mutants. The data were treated as in Fig 1D, S1 Fig and as detailed in the Methods. (C) NON1 and NIA1 expression in 704, non1 and in individual transgenic lines (C1 to C5 and T1 to T9). Transgenics were generated by transforming non1 with a plasmid containing the NON1 genomic sequence. Multiple lines resistant to the antibiotic were selected, and NON1 and NIA1 transcripts were quantified by qRT PCR in each line grown during 6 hours in medium containing NO3−4 mM and NH4+ 4 mM. The five lines that were resistant to the antibiotic but that did not express NON1 were used as negative controls (lines C1 to C5). Two transformants (T8 and T9) were considered false positives because, although NON1 expression was detected in these genotypes, they showed abnormal expression levels of NIA1 and, in the case of T9, of NON1 itself. (PDF) [file pone.0155128.s002.pdf]

**A**

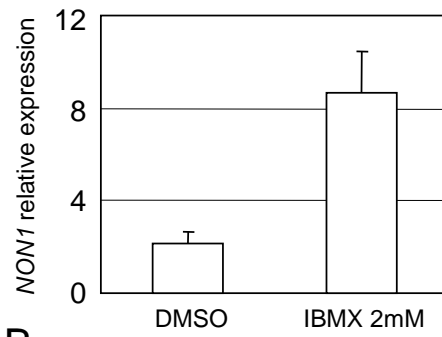

**B**

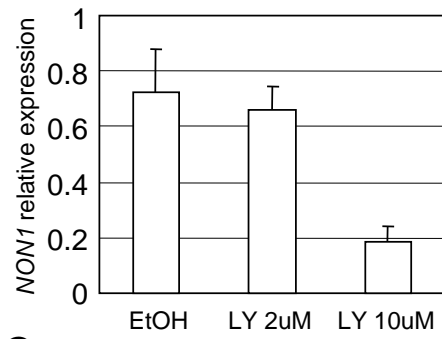

**C**

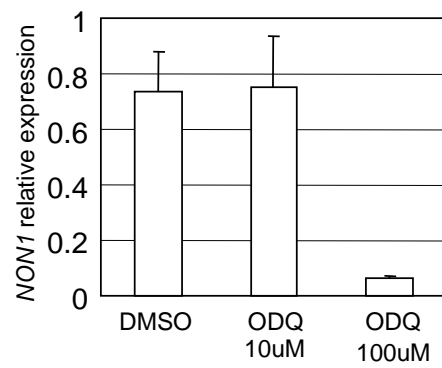

Supplement: S4 Fig — The 704 parental strain was grown on NH4+ 8 mM medium until the cell culture reached exponential phase, and the cells were washed and transferred to media containing (A) NO3- 100 μM or (B) and (C) NO3−4 mM and NH4+ 1 mM. The different chemicals were applied at the indicated concentrations and samples were harvested 1 hour after treatment for quantification of NON1 expression. (PDF) [file pone.0155128.s004.pdf]
