# Supplementary material for: Characterization of a Mutant Deficient for Ammonium and Nitric Oxide Signalling in the Model System Chlamydomonas reinhardtii
Source: PLoS One. 2016 May 5;11(5):e0155128. doi: 10.1371/journal.pone.0155128 (PMC4858171; doi:10.1371/journal.pone.0155128)
Supplement: S1 Table — (DOCX) [file pone.0155128.s005.docx]

**S1 Table. List of primers used in this study.**

| **Gene** | **Primer** | **Sequence** |
| --- | --- | --- |
| *Ubiquitin* (house keeping) | Ubiupper | GTACAGCGGCGGCTAGAGGCAC |
|  | Ubilower | AGCGTCAGCGGCGGTTGCAGGTATCT |
| *20.40CG1* | 20.40RT1fw | GCTGGGACGAGACCATCAAGTGCT |
|  | 20.40RT1rev | GCGGGTCGGGCTGGATGGTG |
| *20.40CG2* | 20.40RT2fw | GGAGGAGCCAACATCAGGGAGAT |
|  | 20.40RT2rev | CCAAAGCACTTGTCGATGAGCAG |
| *CYG56* | 20.73RT1fw | GAGCGCTTCGAGGCAGAGACC |
|  | 20.73RT1rev | CAGCAGTGCGTCAAACTCGTTGTACA |
| *NON1* | 42.49RT1fw | CCTGACGACTCTCCTCTACGGGATG |
|  | 42.49RT1rev | GATAAAGCGGTTCATCCAGTTGTGC |
| *42.49CG1* | 42.49RT2fw | CAAAGCCTTCTACACGCAGGTTCAC |
|  | 42.49RT2rev | CAGCCGCTTGGGGTTCTCACAC |
| *106.20CG1* | 106.20RT1fw | GACCTGAAGATCAAGGTCGAGCACCT |
|  | 106.20RT1rev | CCTCGCCCAGGGTGAACTTGATG |
| *106.20CG2* | 106.20RT2fw | GCTGGGAGTCCGCTGCCTACC |
|  | 106.20RT2rev | CCGTTCTCCAGCTTGCCCACAT |
| *209.82CG* | 209.82RT1fw | ACGTGTTCGGCTGCGAGATCC |
|  | 209.82RT1rev | GCTGCGCACGCTCACCTTCTC |
| *219.8CG* | 219.8RT1fw | CGCTGCGAATGCCAGGCAAT |
|  | 219.8RT1rev | CCTTGAGGCCGGTCCAGTGCA |
| *258.90CG* | 258.90RT1fw | AACGGCGGCCACTTCAGCATC |
|  | 258.90RT1rev | CTGCGCCATCCGTGTGTTGC |
| *259.89CG* | 259.89T1fw | CCTGAGCATTCTGAAGGAGGTGGTG |
|  | 259.89T1rev | CGCCTTGTACTTGGCCGTCTGGT |
| *NIA1* | NRupper | CCGAGCGCTTCCGGCTGTGGTACA |
|  | NRlower | CTGGATCTGGCGGTCCTTGCTGTA |
| *NRT2.1* | Nrt1upper | CGCCGTGGCAACTGACCCTGAG |
|  | Nrt1lower | CGCCACCTCCTCCGCACTCCAC |
| *NIT2* | Tail3N2 | GCGCCATGCGAGGGGTTCACA |
|  | 5RTLowerNit2 | CAGGCTCAAGCTTTTTTTTTTTTTTTGTA |
| *AMT1.1* | Amt1upper | GCACGGGAGGGCAAGAGGTTC |
|  | Amt1lower | ATGTGCCGCAGTCAAGAAGGATTT |
| *AMT1.2* | Amt2upper | GGCTCGCCACCTGCAAGAGACAAC |
|  | Amt2lower | GGCTCGCCACCTGCAAGAGACAAC |
