# Supplementary material for: Characterization of a Mutant Deficient for Ammonium and Nitric Oxide Signalling in the Model System Chlamydomonas reinhardtii
Source: PLoS One. 2016 May 5;11(5):e0155128. doi: 10.1371/journal.pone.0155128 (PMC4858171; doi:10.1371/journal.pone.0155128)
Supplement: S2 Table — (DOCX) [file pone.0155128.s006.docx]

**S2 Table. Details of genes analysed in Fig 1.**

| **Mutants** | **Genes / Candidate genes**  **(analysed for the first time in the current study)** | | | | **Insertions** | |
| --- | --- | --- | --- | --- | --- | --- |
|  | **Name** | **function / domains** | **Reference** | **Position** | **Reference** | **Position** |
| 42.49 / non1 | *42.49CG1 / NON1* | unkown | Cre16.g655050 | XVI: 1770781..1776646 | AY704193 | XVI: 1775804 |
|  | *42.49CG2* | unknown | Cre16.g655100 | XVI: 1776767..1779778 |  |  |
| 85.37 | *85.37CG* | ARID/BRIGHT DNA binding domain / Cytosine specific DNA methyltransferase replication foci domain | Cre12.g508050 | XII: 2239067..2248462 | AY704196 | XII: 2244389 |
| 106.20 | *106.20CG1* | Peptidyl-prolyl cis-trans isomerase, FKBP-type | Cre11.g478750 | XI: 3035123..3038385 | AY704198 | XI: 3038357 |
|  | *106.20CG2* | Peptidyl-prolyl cis-trans isomerase, FKBP-type | Cre11.g478700 | XI: 3032679..3034925 |  |  |
| 209.82 | *209.82CG* | unknown | Cre13.g571400 | XIII: 1331677..1342818 | AY704205 | XIII: 1336765 |

| **Mutants** | **Genes / Candidate genes**  **(described in previous studies)** | | | | **Insertions** | |
| --- | --- | --- | --- | --- | --- | --- |
|  | **Name** | **function / domains** | **Reference** | **Position** | **Reference** | **Position** |
| 20.40 | *20.40CG1* | WD40 repeat-containing protein | Cre12.g552900 | XII: 7860332..7863522 | AY704185 | XII: 7860238 |
|  | *20.40CG2* | Aminomethyltransferase folate-binding domain / Glycine cleavage T-protein C-terminal barrel domain | Cre12.g552850 | XII: 7863654..7869614 |  |  |
| cyg56 / 20.73 | *CYG56* | guanylate cyclase | Cre16.g688901 | XVI: 3707346..3721970 | AY704186 | XVI: 3707940 |
| cdp1 / 219.8 | *CDP1* | cystein rich domain | Cre16.g658800 | XVI: 2232959..2235567 | AY704207 | XVI: 2235177 |
| 258.90 | *258.90CG* | peptidyl-prolyl cis-trans isomerase / cyclophilin-like peptidyl-prolyl cis-trans isomerase family protein | Cre10.g460650 | X: 5693581..5699644 | AY704211 | X: 5696739 |
| 259.89 | *259.89CG* | ubiquitin-conjugating enzyme E2 / sumo-conjugating enzyme UBC9 | Cre01.g019450 | I: 3122836..3125911 | AY704212 | I: 7860238 |

The first part of the table provides information on the six candidate genes whose expression was analyzed and reported for the first time in the current study. The second part of the table provides updated information on the six genes whose expression had previously been described in the same samples used for the experiment presented in Fig 1 (de Montaigu *et al*., 2011). This data was reanalysed and included in the correlation analysis. Physical positions on chromosomes, chromosome numbers, gene reference numbers and functional predictions of genes are based on the data from the latest version of the *Chlamydomonas* *reinhardtii* genome v5.5 (<https://phytozome.jgi.doe.gov/pz/portal.html>). Roman numerals indicate chromosome numbers. The reference numbers of the insertions in each mutant correspond to the Genbank accession number of the genomic sequences flanking the inserts, as previously reported (Gonzalez-Ballester *et al*., 2005).
